# Brief mindfulness-based training and mindfulness trait attenuate psychological stress in university students: a randomized controlled trial

**DOI:** 10.1186/s40359-021-00520-x

**Published:** 2021-02-01

**Authors:** Geovan Menezes de Sousa, Geissy Lainny de Lima-Araújo, Dráulio Barros de Araújo, Maria Bernardete Cordeiro de Sousa

**Affiliations:** Brain Institute, Federal University of Rio Grande do Norte, Av. Sen. Salgado Filho, 3000 - Lagoa Nova, Natal, RN 59078 970 Brazil

**Keywords:** Mindfulness training, Psychological distress, Trait mindfulness, Psychological well-being, Cortisol

## Abstract

**Background:**

Psychological distress in University settings has grown and became a public health concern. In this context, contemplative practices such as mindfulness have been proposed as a strategy to help students on stress management.

**Methods:**

Forty university students (20 female), aged between 18 to 30 years (mean = 24.15; SD = 3.56), with no previous experience with meditation or yoga were recruited at the Federal University of Rio Grande do Norte and randomized to a mindfulness training (MT) or active control (AC) groups. We analyzed measures of anxiety, affect, stress, as well as state and trait mindfulness in order to evaluate the effects of trait mindfulness and a brief mindfulness intervention in forty healthy young students. Participants were classified as Low (n = 27, females = 13) or High (n = 13, females = 7) Trait Mindfulness by k-means clustering and compared between them using Wilcoxon sum rank test. Furthermore, the sample was randomly allocated to an AC (n = 20, females = 10) or a MT (n = 20, females = 10) group, and mixed analysis of variance was performed to analyze the effect of interventions. The mechanisms and role of trait mindfulness in the intervention was assessed by a moderated mediation analysis.

**Results:**

We found that High Trait individuals have lower anxiety trait, anxiety state and perceived stress levels. Only the MT group reduced their anxiety state and perceived stress after the intervention and increased their state mindfulness. Both groups reduced negative affect and cortisol, and no change was found in positive affect. Moderated mediation analysis showed that the training-induced change in state mindfulness mediated the increase in positive affect and the decrease in perceived stress and cortisol, regardless of trait mindfulness. For anxiety state the decrease only occurred in individuals with High Trait Mindfulness.

**Conclusions:**

Together, these results suggest that higher trait mindfulness is associated with low levels of psychological distress and that a brief mindfulness-based intervention seems to be useful to reduce distress measures in university students.

***Trial registration*:**

ReBEC, U1111-1194-8661. Registered 28 March 2017—Retrospectively registered, http://www.ensaiosclinicos.gov.br/rg/RBR-7b8yh8

## Background

Mental health has been a present topic in the university scenario. A survey study published in 2017 pointed out that 12% of PhD students had sought for help with anxiety and depression [1], with a number which increased to an alarming 36% in 2019 [2]. Evidence aiming to uncover the sources of psychological distress among students point to academic-related ones such as their relationship with a supervisor, academic performance and financial concerns [3, 4]. The reported prevalence of major depressive disorder (21.2%) and generalized anxiety disorder (18.6–16.7%) in students is higher than that for the general population (depression, 4.4%; anxiety, 3.6%) [5, 6]. A recent systematic review and meta-analysis of 195 studies accounting for 47 countries showed a prevalence of depressive symptoms around 27.2%, and suicidal ideation of 11.1% in medical students [7]. The high prevalence of psychiatric disorders in this population presents a demand for public policies and strategies which could enhance psychological well-being and help them to cope with adversities, with one example which is under investigation being contemplative practices.

Contemplative practices are becoming even more popular in Western societies, likely because of their potential to improve well-being by several mechanisms [8]. These practices involve a plethora of movement and meditation techniques, with mindfulness meditation being a popular example. Mindfulness is commonly known as a meditation style rooted in Buddhism and can assume a number of definitions not only based on Buddhist principles, but also on psychological and popular conceptions [9]. Herein we make use of its psychological conception of a measurable construct aligned with its Kabat-Zinn’s definition as the act of being present on purpose, acceptance and openness [10]. Mindfulness can also be interpreted as a set of skills composed of state and trait domains [11]. State mindfulness is often increased at the moment and immediately after mindfulness training, while the trait domain is more stable over time but can be enhanced with regular mindfulness training [12]. Both dimensions are measured by self-report instruments such as the State Mindfulness Scale (SMS) for state mindfulness, and the Five Facets of Mindfulness Questionnaire (FFMQ) for trait mindfulness.

Some evidence has pointed to a beneficial effect of high trait mindfulness on mental health and psychological well-being. Tomlinson and colleagues (2018) found a negative relationship of trait mindfulness with depressive and anxiety symptoms, perceived stress and rumination, and a positive relationship with executive functioning and psychological well-being [13]. A recent meta-analysis showed an inverse correlation of trait mindfulness with negative affect symptoms [14]. Importantly, the authors also highlighted the multidimensional nature of trait mindfulness by showing different relationships between FFMQ facets and affective symptoms, in which the Observe facet positively correlated with social anxiety disorder symptoms, the Describe facet negatively correlated with generalized anxiety symptoms, and the Non-react facet inversely correlated with depressive and anxiety symptoms [14]. Furthermore, cross-sectional studies often show a different pattern of brain structure and functioning favoring eudaimonic well-being and receptivity to unpleasant stimuli in individuals with high trait mindfulness [15, 16].

In addition to psychological effects, mindfulness is also reported to modulate physiological measures of stress. Despite evolving as a highly adaptive reaction to homeostasis disruptions, modern lifestyles marked by bad nutrition, poor sleep quality, inactivity and social inequity generally lead to an intermittent and dysregulated stress response [17]. This chronic profile often overcomes beneficial effects of acute stress response and becomes a high-risk factor for developing chronic and mental illnesses [17, 18]. The physiological response to a stressful event is mainly guided by the sympathoadrenomedullary system and hypothalamic–pituitary–adrenal axis through epinephrine and cortisol release, respectively, but is also modulated by the immune and metabolic systems. Signals of stressful events are integrated in the hypothalamus and trigger the release of corticotropin releasing factor by paraventricular nuclei cells, which stimulates the release of adrenocorticotropic hormone by the anterior pituitary. This hormone then stimulates the synthesis and secretion of cortisol by the adrenal glands’ cortices [19]. Regarding the interaction between mindfulness skills and stress mediators, it is hypothesized that higher mindfulness skills buffer stress reactivity, decreasing epinephrine and cortisol responses, and targeting brain areas related to the regulation of these mediators, such as the prefrontal cortex and amygdala [20].

The implication of mindfulness skills and training to improve mental health and psychological well-being has been evaluated by some meta-analyses. Mindfulness-based intervention for psychiatric disorders was found to be better than no treatment and non-active control conditions. Moreover, mindfulness training seems to have a similar effect of evidence-based treatment such as cognitive behavioral therapy and antidepressant medications for depression and anxiety [21], which is in line with other meta-analytic reports [22]. Cultivating mindfulness can not only mediate changes in mental health and well-being by increasing mindfulness skills, but also by reducing repetitive negative thinking, such as rumination [23]. In addition to psychological effects, meditation training also affects biological measures of stress response [24]. The authors evaluated markers of neuroendocrine, immune and autonomic systems of 45 randomized controlled trials and found a high evidence level of reductions in plasma cortisol and resting heart rate, and a moderate level for C-reactive protein, TNF-α, blood pressure and triglycerides, thus favoring meditation practice over active interventions such as exercise and relaxation [24], and suggesting a diffuse systemic effect of meditation which could provide some protection against dysregulation of stress response systems.

In university settings, a growing body of evidence is showing that mindfulness training leads to improvement in several well-being measures. A recent large prospective study showed that 8 weeks of mindfulness course reduced psychological distress and improved well-being during and after an examination period, which is a significant source of stress for students [25]. The same intervention time reduced mental distress and study-related stress, while also increasing subjective well-being [26]. In addition to long-term interventions, there are also some reports that brief mindfulness interventions improve mood and decrease stress in academic and scholar settings [27, 28]. In addition to mindfulness training, trait mindfulness also seems to play a role, as it has been reported to mediate the development of empathy in university students [29].

In this context, we make use of an exploratory approach in order to investigate whether trait mindfulness influences baseline measures of well-being in non-meditators and to analyze changes in affection, anxiety and cortisol levels induced by a brief mindfulness training (defined as up to one-week multiple sessions of brief mindfulness meditation [30]), as well as the possible role of trait mindfulness in their expression. Our hypotheses are threefold: first, higher trait mindfulness is related to psychological well-being in an inverse relationship with measures of anxiety, stress and negative affect, and directly related to positive affect; second, brief mindfulness training reduces negative indicators of psychological well-being while increasing the positive ones; and finally, that the increase in positive affect and decrease of cortisol and negative outcomes (state anxiety, negative affect and perceived stress) are mediated by meditation induced elevation in state mindfulness and moderated by trait mindfulness.

## Methods

### Experimental design and interventions

**Fig. 1:**
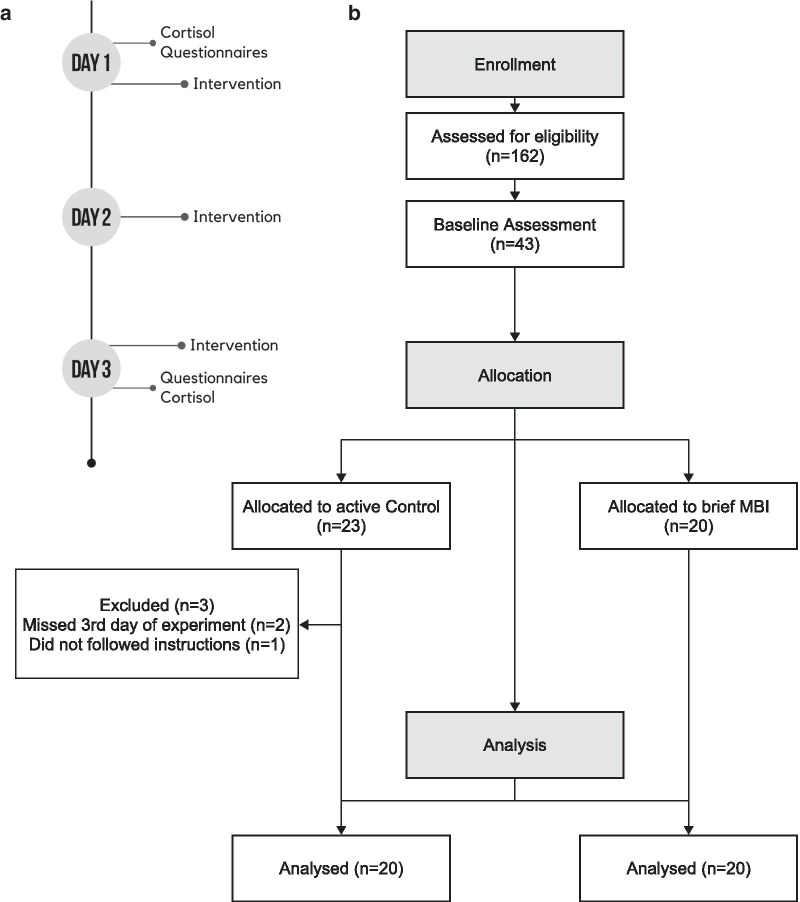
Experimental design (**a**) and CONSORT (Consolidated Standards of Reporting Trials) study flow (**b**). Forty-three participants were allocated (Control: n = 23, Mindfulness: n = 20) but 3 excluded from Control, remaining 20 individuals per group. MBI = Mindfulness-Based Intervention

The sample consisted of graduate and undergraduate students (mean age: 24.15 ± 3.61) of the Federal University of Rio Grande do Norte enrolled by online recruitment (2017–2018) and were eligible if they declared the absence of: psychiatric disorders, psychotropic or anti-inflammatory prescriptions and had experience with meditation or yoga. Age- and gender-matched participants were allocated by simple randomization to either a mindfulness training (MT group, n = 20, females = 10, mean age: 24.05 ± 3.76) consisting in an audio-guided meditation focused on the body and breathing sensations, or to an active control group (AC, n = 20, females = 10, mean age: 24.25 ± 3.55), characterized by coloring pictures and listening to an audio related to health themes. Sample size was determined with G*Power from F tests family (RM-ANOVA with within-between interaction), considering a moderate to high effect size (f = 0.3) at α = 0.05 and 80% of power, as well as 60% of correlation between repeated measures. The study was a parallel trial and occurred during three consecutive days at the Brain Institute’s Laboratory of Encephalography. The interventions lasted for 30 min a day, totalizing 90 min of intervention for each group. The active control was introduced to two activities: listening to audio containing educational health information for about 15 min, and then the participants were asked to color pictures to fill the time in order to remain active for the same time as the mindfulness training group (30 min a day). Regarding the mindfulness training group, during the 3 days of intervention, the same 30-min audio for meditation practice was played. The audio recording of the mindfulness group was derived and adapted from a standard mindfulness sitting meditation practice and the script is available upon request. Self-reported psychometric instruments (see Sect. 2.2) and blood samples were collected on the first and third days before (on the first day) or after (on the third day) intervention (Fig. 1a). Eligibility was reached by 162 students, but only 40 were allocated and analyzed (MT = 20, females = 10; AC = 20, females = 10) (Fig. 1b). All procedures were leaded by GLLA and were approved by the ethical committee of the Federal University of Rio Grande do Norte, Brazil (CAEE.55193416.4.0000.5537, 1.761.383).

### Questionnaires and hormonal assessment

State and trait mindfulness were assessed by the State Mindfulness Scale (SMS) and Five Facets of Mindfulness Questionnaire (FFMQ), respectively. SMS is a 5-point Likert scale which assesses state mindfulness in the moment of response [31]. FFMQ is a 5-point Likert questionnaire composed by five facets of mindfulness skills, namely Observing (noticing to internal and external experiences), Describing (labeling internal experiences), Act with Awareness (focusing on activities of the moment, as opposed to automatic pilot), Non-judgment of inner experience (non-evaluative stance about thoughts and feelings) and Non-reactivity to inner experience (allow thoughts and feelings to come and go) [32, 33]. Internal consistency (Cronbach’s alpha, α) for these instruments were good for our sample (SMS: α = 0.91, FFMQ: α = 0.85). State and trait anxiety were measured by the 4-point Likert State and Trait Anxiety Inventory (STAI), composed of two parts: the State Anxiety Inventory (SAI, α = 0.90) and Trait Anxiety Inventory (TAI, α = 0.81) [34, 35]. The Positive and Negative Affect Schedule (PANAS) was used to evaluate positive (PAS, α = 0.87) and negative affect (NAS, α = 0.84) [36, 37]. Perceived stress was assessed by the 14-question version of the Perceived Stress Scale (PSS, α = 0.83) [38, 39].

Blood samples (10 mL) were collected by venipuncture on the 1^st^ and 3^rd^ days of training between 8:00 AM and 9:00 AM after 45 min of resting. Participants were requested to avoid caffeine consumption on the 1st and 3rd days of the experiment. Plasma cortisol was measured by chemiluminescence (Access Cortisol, Beckman Coulter, Cat. Number 33600). Intra-assay coefficient of variation was 4.39 ± 5.92%.

### Data mining and analysis

#### Classification of mindfulness trait

A clustering analysis was implemented in order to classify the sample based on trait mindfulness levels, using the baseline scores of FFMQ facets as grouping variables (Describe, Act with Awareness, Non-judge and Non-react). Despite the good internal consistency of FFMQ, this instrument has some particularities, mainly when using it with a mixed sample of meditators and non-meditators [40]. Specifically, the Observe facet is sensitive to experience with meditation, in which it fits well in confirmatory analysis when the sample has some experience with meditation, but not in an overall sample [32, 40]. These data suggest that a four-factor structure of this questionnaire may be more reliable when dealing with non-meditators, leading to the exclusion of the Observe score when using total FFMQ [9, 32]. Thus, this facet was not included as a grouping variable since all of our sample comprised non-meditators.

The number of clusters was calculated based on the several indexes provided by the *NbClust* package available in R software [41] and this value was then used to compute k-means. The k-means method is an unsupervised learning algorithm used to classify non-previously labelled data. It works by randomly and repeatedly positioning k centers to the n-dimensional scatter plot and calculating the distance between each point of the data from these centers until the assignment of centers do not change (the so-called iteration process). We used Euclidean distance to calculate similarity between centers and 25 as the minimal number of iterations. Wilcoxon sum rank test was used to compare facets between clusters.

#### Baseline and post-intervention comparisons

Baseline measures of perceived stress, positive and negative affect, anxiety trait and state, state mindfulness and cortisol were compared across clusters using Wilcoxon sum rank test. We performed mixed analysis of variance (ANOVA) to assess the effect of intervention on these measures, using group as the between factor and session (pre and post intervention) as the within factor. Investigation of potential influences of trait mindfulness on intervention outcomes is described in the next section. We adjusted p-values for each repeated variable tested based on Benjamini and Hochberg method (False Discovery Rate) in order to avoid inflation of type I error due to multiple testing, which provides an interesting approach since its adjustment not only reduces false positives, but also false negatives [42]. Effect sizes are given by Cohen’s *d* for mixed ANOVA and $|r|$ for Wilcoxon sum rank tests. The analyzes were performed using R software.

#### Mechanisms of brief mindfulness intervention and influence mindfulness trait

We used second-stage moderated mediation in order to have some exploratory insight into mechanisms of the brief mindfulness intervention, since it is a potentially powerful analysis and commonly used in psychological research [43, 44]. Mediation analysis tests if the effect of an independent variable X on a dependent variable Y (X → Y) is mediated by a variable M (X → M, a-path; M → Y, b-path). Mediation effect occurs when there is a significant indirect effect (X → M → Y, both a- and b-path is significant), and the effect of X → Y controlling for the indirect effect is lost or decreased (so-called c’-path or direct effect). When the significance of c’-path is lost it is called full mediation, while it is called partial mediation when it remains significant (but with a significant indirect effect). Second-stage moderated mediation tests if a mediation effect is moderated by a variable W specifically on b pathway, which means that if the effect of the mediator variable on an outcome occurs at certain levels of moderator (W). The presence of moderated mediation can be assessed by the index of moderated mediation (ω), defined as

$$\omega = a\left( {b_{1} + b_{3} W} \right),$$

where *a* stands for estimated a-path, *b*_*1*_ stands for the estimated effect of the mediator on the dependent variable in the absence of the moderator, and *b*_*3*_*W* stands for the estimated interaction effect between the mediator and moderator on the dependent variable [45]. The index of moderated mediation is considered significant when its bootstrapped confidence interval (95%) does not cross zero, and can be interpreted in terms of modulation of indirect effect. In other words, that when ω > 0, the indirect effect increases (i.e. the mediation effect gets stronger), as opposed to when ω < 0. Herein, we used the intervention group as the independent variable (X, AC = 0, MT = 1), state mindfulness as the mediator (M), variables which changed within MT after intervention as outcomes (Y), and the trait mindfulness level as the moderator (W). We used the difference between scores of the 3rd and 1st days (Δ = Post – Pre) of mediator and dependent variables. Indirect effects were estimated with 10,000 bootstrap samples. The analysis was performed using the *processr* package (version 0.0.0.9000; *lavaan* package version 0.6.5) available in R software.

All analyses were performed using RStudio Integrated Development Environment (version 1.2.5033) for R software (version 3.6.1). Statistical significance was set to *p* ≤ 0.05 (two-tailed). When 0.05 < *p* ≤ 0.10, we refer to it as marginal significance or a trend. The confidence interval (95%) is reported inside brackets as [lower limit, upper limit].

## Results

### Definition of mindfulness trait

**Fig. 2:**
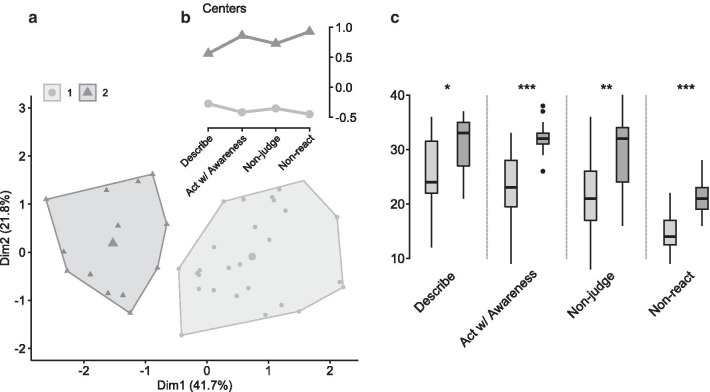
Cluster plot showing the two dimensions with higher variability (**a**) and centers of grouping variables for each cluster (**b**), showing a clear separation between individuals with low (Cluster 1) from those with high mindfulness trait (Cluster 2). (**c**) Boxplots of FFMQ facets. Wilcoxon sum rank test showed that all facets are significantly different between clusters. *p ≤ .05, **p ≤ .01, ***p ≤ .001

The majority of indexes provided by the *NbClust* package suggested 2 as an optimal number of clusters and therefore that was the number provided to k-means. The overall variability on the two dimensions of the cluster accounted for 63.5% (Fig. 2a). The clusters were labelled as Low Trait (Cluster 1, n = 27) based on the centers (weight of each variable in the two cluster dimensions) of grouped variables, since its centers were below the mean, and High Trait (Cluster 2, n = 13) due to its high centers (Fig. 2b). All facets showed to be different between clusters (Wilcoxon sum rank test: Describe: W = 88.5, *p* = 0.01, p-adjusted = 0.03, r = 0.40; Act with Awareness: W = 38.5, *p* = 7.53e−05, p-adjusted = 0.0003, r = 0.63; Non-judge: W = 72.5, *p* = 0.003, p-adjusted = 0.09, r = 0.47; Non-reactivity: W = 36, *p* = 5.65e−05, p-adjusted = 0.0001, r = 0.64), therefore indicating good segregation (Fig. 2c). Cluster validation metrics can be accessed in Additional file 1. Variables means and standard deviations across trait mindfulness levels can be seen in Table 1. There was equity in sex distribution into each cluster (Additional file 2).

**Table 1:** Mean and standard deviation (SD) of variables between mindfulness trait levels

	Low trait		High trait		*p *value	Effect size (r)
	Mean	SD	Mean	SD		
Age	23.93	3.51	24.62	3.93	.52	.10
Describe	25.30	7.15	31.23	5.26	**.01**	**.40**
Act w/ awareness	23.48	6.31	32.23	3.24	** < .0001**	**.63**
Non-judge	21.22	6.41	29.46	7.11	**.003**	**.47**
Non-react	14.96	3.52	21.31	3.57	** < .0001**	**.64**
State mindfulness	71.93	11.55	75.23	10.87	.32	.16
Positive affect	28.93	6.62	30.85	8.35	.59	.09
Negative affect	24.96	7.05	20.31	6.02	.08	.28
Anxiety state	41.15	8.69	35.08	5.41	**.01**	**.39**
Anxiety trait	49.30	6.78	40.92	7.04	**.001**	**.51**
Perceived stress	32.52	6.61	26.46	6.28	**.01**	**.40**
Cortisol	10.38	3.96	13.09	4.98	.07	.28

Bold values indicate significant comparisons

### Baseline measures of well-being between levels of mindfulness trait

**Fig. 3:**
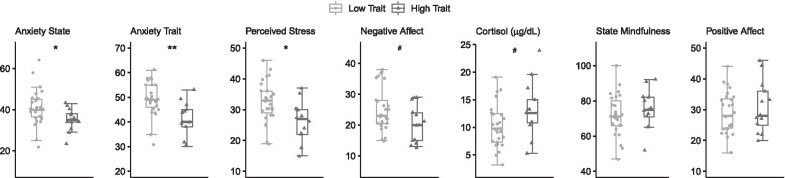
Well-being measures before intervention between low and high mindfulness trait. For means and standard deviations, see Table 1. Wilcoxon sum rank test, #*p* < .10, *p < .05, **p < .01

Wilcoxon sum rank test found that Anxiety State (W = 261, *p* = 0.01, p-adjusted = 0.02, r = 0.39), Anxiety Trait (W = 287, *p* = 0.001, r = 0.51) and Perceived Stress (W = 264, *p* = 0.01, p-adjusted = 0.02, r = 0.40) were significantly lower in High Trait when compared with Low Trait individuals. A trend in High Trait individuals in showing lower levels of Negative Affect (W = 237, *p* = 0.08, p-adjusted = 0.08, r = 0.28) and higher levels of Cortisol (W = 113, *p* = 0.07, p-adjusted = 0.07, r = 0.28) were also found. No difference was found for State Mindfulness (W = 141, *p* = 0.32, p-adjusted = 0.32, r = 0.16) or Positive Affect (W = 156.5, *p* = 0.59, p-adjusted = 0.74, r = 0.09) (Fig. 3, Table 1). A plot summarizing the between groups effect size for each variable can be seen in red in Additional file 3.

### Well-being measures post-intervention within and between AC and MT groups

**Fig. 4:**
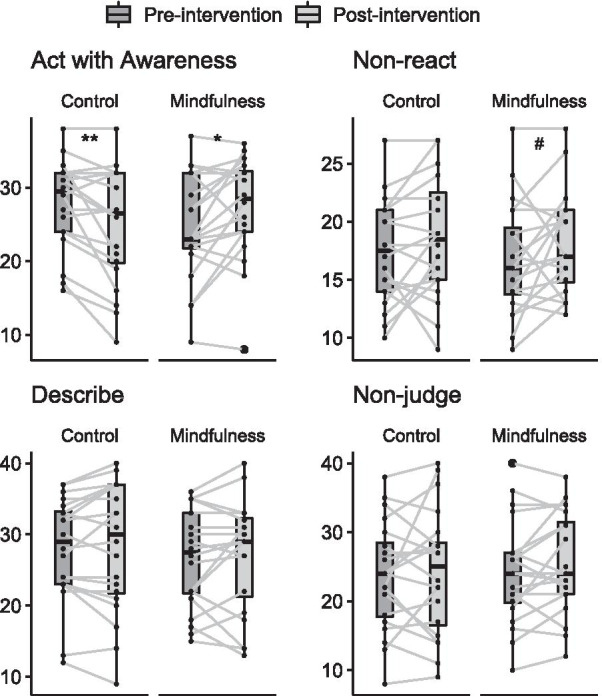
Facets of FFMQ before and after interventions for each group. For means and standard deviations, see Table 2. Mixed ANOVA test, #*p* ≤ .10, *p ≤ .05, **p ≤ .01, ***p ≤ .001

For the FFMQ facets, the Mixed ANOVA showed an interaction between groups and session for the Act with Awareness facet (Group*Session: F_(1,38)_ = 15.00, *p* = 0.0004) with a decrease for the control group (AC: Post – Pre ± Standard Error =− 3.00 ± 1.03, *p* = 0.006, p-adjusted = 0.009, *d* = 0.72) and an increase for the mindfulness group (MT: Post–Pre = 2.65 ± 1.03, *p* = 0.01, p-adjusted = 0.01, *d* = 0.53), as well as a significant session effect for the Non-react facet (Session: F_(1,38)_ = 4.62, *p* = 0.03; Post– Pre = 1.3 ± 0.60), where only an increasing trend in the mindfulness group was found (MT: Post–Pre = 1.45 ± 0.85, *p* = 0.098, p-adjusted = 0.14, *d* = 0.35; AC: Post – Pre = 1.15 ± 0.85, *p* = 0.19, p-adjusted = 0.19, *d* = 0.35) (Fig. 4, Table 2).

**Table 2:** Mean and standard deviation (SD) of variables between groups

		Control			Mindfulness		
		Mean	SD	*d*	Mean	SD	*d*
Age		24.25	3.55	-	24.05	3.76	-
Describe	Baseline	27.90	7.33	.11	26.55	6.99	.14
	Post	28.25	9.08		27.10	7.88	
Act w/ awareness	Baseline	27.95	6.02	**.72**	24.70	7.39	**.53**
	Post	24.95	7.87		27.35	6.96	
Non-judge	Baseline	23.65	7.90	.09	24.15	7.55	.32
	Post	24.20	9.12		25.50	7.31	
Non-react	Baseline	17.40	4.47	.34	16.65	4.84	.34
	Post	18.55	5.15		18.10	4.39	
State mindfulness	Baseline	74.50	13.02	.35	71.50	9.38	**.73**
	Post	69.95	17.54		76.70	12.09	
Positive affect	Baseline	30.90	7.43	.07	28.20	6.83	.40
	Post	30.55	5.92		30.20	7.40	
Negative affect	Baseline	24.75	8.23	**.97**	22.15	5.43	**.87**
	Post	19.90	7.26		17.70	5.97	
Anxiety state	Baseline	38.65	9.43	.00	39.70	7.03	**.57**
	Post	38.65	9.82		35.20	7.28	
Perceived stress	Baseline	30.50	7.39	.45	30.60	6.85	**.93**
	Post	28.35	6.65		24.25	8.18	
Cortisol	Baseline	11.53	5.18	**.57**	10.99	3.68	**.56**
	Post	9.71	5.35		9.81	3.78	

Bold values indicate significant comparisons. *d* = Cohen’s d effect size

**Fig. 5:**
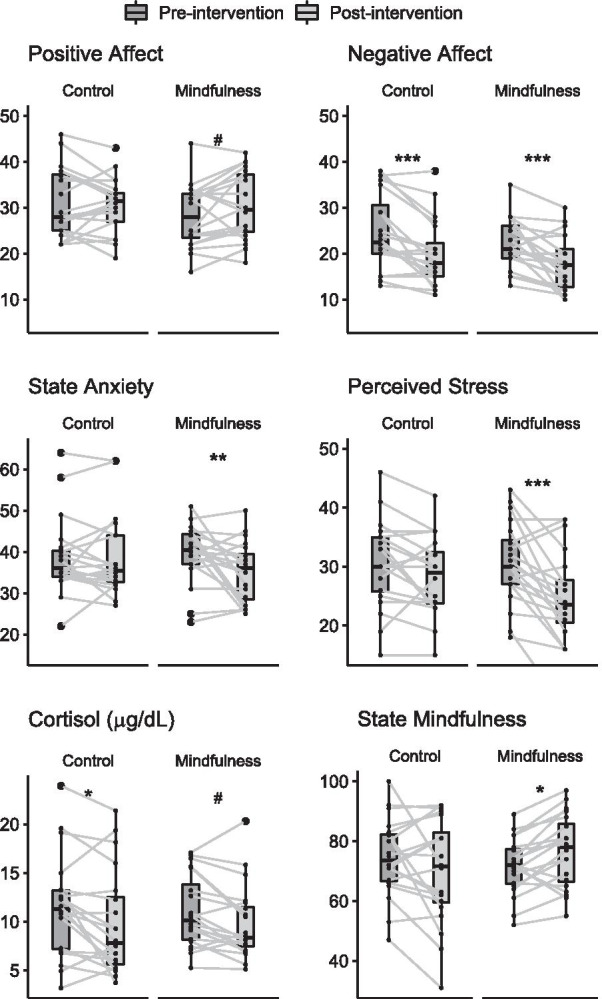
Well-being measures before and after interventions for each group. Mindfulness training induced an overall change in psychological and physiological variables. For means and standard deviations, see Table 2. Mixed ANOVA test, #*p* ≤ .10, *p ≤ .05, **p ≤ .01, ***p ≤ .001

For well-being measures, the Mixed ANOVA showed session effects for all variables (Negative Affect: F_(1,38)_ = 33.76, *p* = 0.000001, Post – Pre = − 4.65; Anxiety State: F_(1,38)_ = 4.34, p = 0.04, Post – Pre = − 2.25; Perceived Stress: F_(1,38)_ = 33.76, p = 0.00005, Post – Pre = − 4.25; Cortisol: F_(1,38)_ = 12.32, *p*= 0.001, Post – Pre = − 1.5), except for Positive Affect (Session: F_(1,38)_ = 1.16, p = 0.28) and State Mindfulness (Session: F_(1,38)_ = 0.03, p = 0.84). Interactions between group and session were found for Anxiety State (Group*Session: F_(1,38)_ = 4.34, p = 0.04), Perceived Stress (Group*Session: F_(1,38)_ = 5.14, p = 0.03) and State Mindfulness (Group*Session: F_(1,38)_ = 8.63, p = 0.005), where only mindfulness training reduced both Anxiety State (MT: Post – Pre = − 4.5, p = 0.005, p-adjusted = 0.01, *d* = 0.57; AC: Post – Pre = 0.0, *p* = 1.00, p-adjusted = 1.00, *d* = 0.00) and Perceived Stress (MT: Post – Pre = − 6.35, p = 0.00001, p-adjusted = 0.00003, *d* = 0.93; AC: Post – Pre = − 2.15, p = 0.11, p-adjusted = 0.11, *d* = 0.45), while State Mindfulness increased (MT: Post – Pre = 5.20, p = 0.03, p-adjusted = 0.09, *d* = 0.73). The AC group showed a decreasing trend in State Mindfulness (AC: Post – Pre = − 4.55, p = 0.06, p-adjusted = 0.09, *d* = 0.35). Both groups reduced Negative Affect (MT: Post – Pre = − 4.45, p = 0.0003, p-adjusted = 0.0004, *d* = 0.87; AC: Post – Pre = − 4.85, p = 0.0001, p-adjusted = 0.0003, *d* = 0.97). AC significantly reduced Cortisol (AC: Post – Pre = − 1.83, p = 0.005, p-adjusted = 0.01, *d* = 0.57), while a marginal decrease was found for MT (MT: Post – Pre = − 1.18, p = 0.058, p-adjusted = 0.07, *d* = 0.56) (Fig. 5, Table 2). A summary of the within effect sizes can be seen in Additional file 3 for both AC (blue) and MT (green).

### Assessment of state mindfulness-induced mechanisms and role of mindfulness trait

We performed a second-stage moderated mediation in order to evaluate whether trait mindfulness has some role in brief mindfulness training-induced changes on measures of well-being. We used those variables that significantly or marginally changed after mindfulness training as outcomes, state mindfulness as the mediator and the trait mindfulness level as the moderator.

**Fig. 6:**
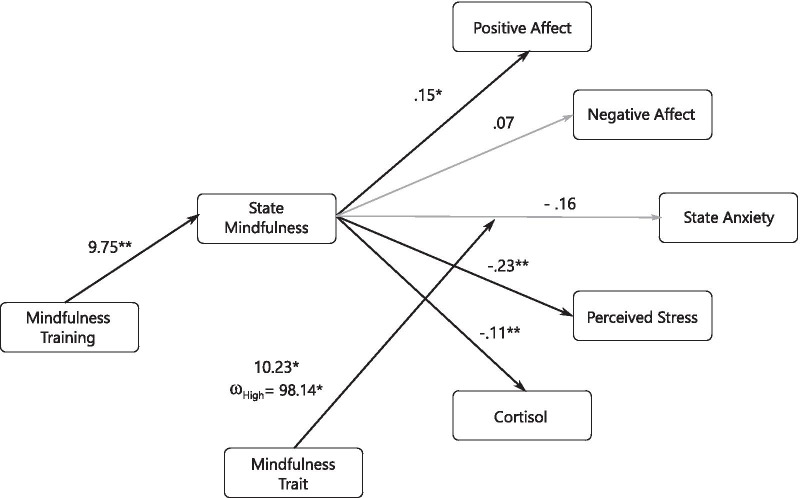
Conceptual diagram of second-stage moderated mediation showing overall mediation effect of mindfulness training and the role of high mindfulness trait as moderator of mediation effect of State Mindfulness on Anxiety State. Values on State Mindfulness → Outcomes pathways indicate b_1_ estimate, while that on Mindfulness Trait → Anxiety State indicate b _3_ estimate and Index of Moderated Mediation for High Mindfulness Trait condition (ω_High_). Black arrow stands for significant path and grey line stands for no significant path. For ease of presentation c’-path is omitted, see Table 3 for indexes of moderated mediation, bootstrapped confidence interval and c’-path. *p ≤ .05, **p ≤ .01

A significant a-path showed that mindfulness training increased state mindfulness (a = 9.75,  *p* = 0.003). A full (but not moderated) mediation effect was found for state mindfulness increasing positive affect (b_1_ = 0.15 [0.03, 0.27], p = 0.01) and decreasing perceived stress (b_1_ =− 0.23 [− 0.37, − 0.09], *p* = 0.001) and cortisol (b_1_ = − 0.11 [- 0.20,− 0.03], *p* = 0.007). No overall mediation effect was found for negative affect (b_1_ = 0.07, *p* = 0.36) (Fig. 6, Table 3). Trait mindfulness partially moderated the decrease in anxiety state (b_3_ = 10.23, *p* = 0.005, ω = 99.70 [26.33, 234.58]), showing that the mediation effect gets stronger when trait mindfulness is high (ω_High_ = 98.14 [25.41, 231.62], ω_Low_ = − 1.56 [− 4.72, 0.43]) (Fig. 6, Table 3). In other words, State Mindfulness only mediates the decrease in anxiety under the condition of High Trait Mindfulness.

**Table 3:** Pathways and conditional effects of second-stage moderated mediation

	a	b_**1**_	b_**3**_	c’	ω	ω_**Low**_	ω_**High**_
PAS	9.75	**.15**	− 5.55	3.19	− 54.15	1.50	− 52.65
Boot CI	3.61, 16.45	**.03, .28**	− 11.33, 4.46	− .42, 6.25	− 145.76, 23.79	.24, 3.79	− 143.12, 25.24
NAS	9.75	.07	− 2.22	.25	− 21.63	.71	− 20.92
Boot CI	3.60, 16.45	− .11, .21	− 15.77, 5.54	− 3.00, 3.59	− 179.23, 51.76	− .86, 2.50	− 176.45, 53.02
SAI	9.75	− .16	**10.23**	− **5.50**	**99.70**	− 1.56	**98.14**
Boot CI	3.58, 16.56	− .34, .08	**2.95, 117.25**	− **10.51, **− **.58**	**26.33, 234.58**	− 4.72, .44	**25.41, 231.62**
PSS	9.75	− ** .23**	2.00	− 1.92	19.50	− 2.24	17.27
Boot CI	3.64, 16.57	− ** .37, **− **.09**	− 4.28, 7.87	− 6.44, 2.57	− 39.98, 93.24	− 4.57, − .67	− 42.80, 88.93
Cortisol	9.75	− ** .11**	2.99	1.12	29.20	− 1.12	28.08
Boot CI	3.67, 16.59	− ** .20,− .03**	− 1.01, 6.42	− .77, 2.84	− 3.76, 83.57	− 2.20, − .41	− 4.38, 82.00

a = Mindfulness Training → State Mindfulness pathway, b_1_ = State Mindfulness → Outcome pathway, b_3_ = interaction term between b_1_ and Mindfulness Trait, c’ = Mindfulness Training → Outcomes pathway after removing contribution of mediator, ω = Index of Moderated Mediation, ω_Low_ = Index of Moderated Mediation in Low Mindfulness Trait condition, ω_High_ = Index of Moderated Mediation in High Mindfulness Trait condition, PAS = Positive Affect Schedule, NAS = Negative Affect Schedule, SAI = State Anxiety Inventory, TAI = Trait Anxiety Inventory, PSS = Perceived Stress, Boot CI = bootstrapped 95% confidence interval. Significant estimates are shown in bold

## Discussion

Mindfulness-based practices have been used as a complementary approach to address stress-related issues like anxiety and depression [46], being increasingly used in University settings [25, 26, 47]. Moreover, little is known about the impact of trait mindfulness on well-being measures before mindfulness training. Our aims in this study were mainly twofold: to assess the influence of trait mindfulness level on baseline well-being measures and to evaluate the effects of a brief mindfulness training to account for trait mindfulness on these outcomes, using a sample of graduate and undergraduate students.

According to our results, there is a marked relation between trait mindfulness and measures of well-being such as affect, perceived stress and anxiety. Our data shows that individuals with higher trait mindfulness have low levels of stress, anxiety state and anxiety trait, supporting our first hypothesis. After a brief mindfulness training we observed a decrease in negative affect, anxiety state, perceived stress and a marginal decrease in cortisol and increase in positive affect, corroborating our second hypothesis. We also found a mediation effect of meditation on positive affect, perceived stress and cortisol through state mindfulness. However, the mediation effect for anxiety state only occurred in High Trait individuals, partially confirming our third hypothesis.

There are plenty of ways to categorize different groups based on trait mindfulness such as by median split [48] or by latent profile analysis, a finite gaussian mixture model [49, 50]. Classification by latent profile analysis applied to college students often shows a 4-profile solution [49, 50]. Specifically, in addition to the two “extreme” profiles (all high and all low scores on trait mindfulness facets), the authors found two intermediate profiles called “judgmentally observing”, showing a high score in the Observe facet and a low score in Non-judge and Act with awareness facets, and the “non-judgmentally aware” profile due to its high score in Non-judge and Act with awareness and low Observe score. Importantly, good psychological health outcomes were found for “non-judgmentally aware” and “high mindfulness” profiles and poor psychological health for “judgmentally observing” and “low mindfulness” [49, 50]. Interestingly, the adaptive profiles do not differ between them, while the maladaptive profiles do not differ in almost all outcomes.

We used an unsupervised learning algorithm (k-means) in the present study to label the sample in terms of trait mindfulness level. Classification was based on data collected from the FFMQ instrument without the Observe facet due to its unreliability in a non-meditator population [32]. According to indexes which provide the better number of clusters, our sample was better categorized on two subsamples that comprised individuals who score high in all mindfulness facets and those who score low in all facets. In this sense, we found that High Trait individuals score lower in measures of psychological distress such as anxiety and perceived stress, and marginally in negative affect. So, it seems that removing Observe from the grouping techniques collapses the intermediate profiles found by the latent profile analysis for college students [49, 50]; specifically, the “non-judgmentally aware” profile would group with the High Trait cluster, while the “judgmentally observing” profile would group with the Low Trait cluster, which would explain the similar psychological health pattern shared by these profiles and our clusters.

The relationship between trait mindfulness and psychological health has been quite reported [13]. Executive functioning seems to be positively correlated with trait mindfulness dimensions such as Act with Awareness and Non-judge in a student sample [51]. Additionally, high trait mindfulness has a protective function against rumination and depressive symptoms [52, 53]. Indeed, indirect effects of the Non-judge, Non-react and Describe facets in reducing negative affect and physiological arousal while increasing positive affect occur through a decrease in rumination levels [54]. Similarly, a recent correlational meta-analysis showed that both total FFMQ scores and its Act with awareness, Non-judge, Non-react and Describe facets are negatively correlated with affect symptoms related to anxiety, depressive and post-traumatic stress disorders [14].

Regarding stress response, there are some studies showing that trait mindfulness improves emotional regulation and buffers against the negative influence of perceived stress (for review, see [13]). This might explain the lower perceived stress in the high trait mindfulness group, even with marginally higher cortisol levels as shown by our data. Interestingly, no difference was found for state mindfulness between Low and High Trait, which would seem paradoxical since state is expected to parallel trait. The reason for this incongruence may be explained by the temporally and context sensitive psychometric properties of the State Mindfulness Scale [31, 55]. This scale was precisely designed to catch the perceived awareness of an experience after a mindfulness practice or some other activity during a specific period. Therefore, it is possible that a non-meditator baseline self-reporting of this scale could potentially mismatch with trait mindfulness, even for those categorized as having high trait mindfulness.

We found that mindfulness training significantly reduced negative affect, anxiety state, and perceived stress, while it increased state mindfulness, and marginally decreased cortisol and increased positive affect. Interaction between groups and session showed interesting results pointing that only the mindfulness group significantly changed anxiety state, perceived stress and state mindfulness. An increase in state mindfulness after brief mindfulness training is often reported [56, 57], but not for a very brief intervention, such as a single 15-min training session [58]. The effect of mindfulness training on affective measures is a common report [58–60].

In turn, the control group significantly reduced negative affect and decreased cortisol, which points to a possible psychophysiological effect of a simple painting activity; moreover, the presence of a placebo effect summed with relaxation promoted by painting is not ruled out. In fact, relaxing activities have shown to decrease cortisol levels [61, 62]. Moreover, the painting activity does not seem to have an effect on state mindfulness and anxiety, which corroborates with another study recently published showing that painting/coloring itself has no impact unless it is executed with mindfulness instructions [63].

Although there is no group effect on cortisol levels, the control group showed a slightly greater decrease in cortisol levels after the intervention than mindfulness group (Control: d = 0.57; Mindfulness: d = 0.56). Cortisol is an important energy regulatory hormone and there is evidence suggesting that it modulates attentional processing [64], and facilitates emotional regulation [65]. Engagement is an activity that requires attentional resources, as occurs in mindfulness practices, and may prevent the reduction of cortisol, especially in an untrained population. It is important to note that, when engaging in meditative practices, novice meditators experience higher levels of cognitive effort when compared to expert meditators [66]. Thus, taking into account that blood samples were collected shortly after the intervention, on the third day of the experiment, a slight increase in cortisol related to the attentional process may have been captured, masking the possible decrease induced by the training, which was expected to be more pronounced in the mindfulness group. The active control group, in turn, received no instructions in this regard, and the decrease in cortisol probably did not change after completing the task in this group.

We also found that the increase in state mindfulness induced by meditation training fully mediated the increase in positive affect and the decrease in perceived stress and cortisol. It is known that state mindfulness progressively predicts higher levels of positive affect and lower levels of negative affect [67, 68], but its reported mediation effect is often related to cognitive reappraisal and insight problem-solving [69, 70]. Although there is an inverse relationship between state mindfulness and stress measures such as cortisol and perceived stress [24, 71], no mediating effect of brief mindfulness through state mindfulness on these measures were reported. Other commonly reported mediators of mindfulness training are rumination and trait mindfulness; in these instances, mindfulness skills reduce rumination and boost trait mindfulness, thereby increasing well-being. Maybe these other mediators of mindfulness training are influenced by state mindfulness throughout practice and its mediation effect is somehow due to the increase in state mindfulness. This hypothesis could be further investigated using the conditional processes approach, for instance implementing mediated mediation analysis [72].

We additionally showed that trait mindfulness moderated the indirect effect of mindfulness training on anxiety state and trait through state mindfulness. This effect importantly only occurs in a high trait mindfulness condition. Even though we report large confidence intervals, suggesting a small effect, it still points to an important role of trait mindfulness on anxiety which should be addressed in future studies and considered when designing interventions to study anxiety measures. In a study using first-stage moderated mediation to investigate the influence of trait mindfulness to buffer incivility-stress relation while strengthening incivility-forgiveness relation as mediated by rumination and negative affect [73], the authors found that the ability to incivility to lead to stress through rumination and negative affect is buffered by high levels of Describe and Act with Awareness facets, respectively. Moreover, the indirect effect of incivility on forgiveness through rumination and negative affect only occurs at moderate and low levels of Describe and Non-judge facets, respectively [73]. It supports a role of trait mindfulness in buffering stress, while promoting forgiveness in a multidimensional way.

This study presented some key limitations. First, we highlight its exploratory nature, with its main weakness being type I error inflation due to multiple testing with the same sample. We have tried to minimize it by implementing a p-value correction for each variable across testing. Secondly, our small sample underpowered statistical tests, which made it difficult to capture subtle effects. Therefore, we reinforce the need for more studies using larger sample sizes to avoid type II error. A one-point blood sample does not seem to be sufficient to clearly describe the dynamics of the regulatory response exerted by the HPA axis. Thus, we indicate two possibilities, the increase in the number of sampling in the first hour after awakening (n = 3; cortisol awakening curve) or throughout the day (n = 4; diurnal cortisol curve), in order to clarify the interpretation of the findings on cortisol. In addition, we suggest future studies to conduct a follow-up to better understand the time-varying effects of brief mindfulness interventions. Nevertheless, our study has led to important findings towards understanding the acute psychophysiological effects of a brief mindfulness training and, to our knowledge, this is the first to target the interaction between state and trait mindfulness using moderated mediation analysis, constituting a useful tool for studying psychophysiological mechanisms induced by psychological therapies.

## Conclusions

Together, our results point to interesting findings which place brief mindfulness interventions and trait mindfulness as factors to be, respectively, cultivated and a goal to be developed by university students to reduce stress-related symptoms and potentially protect against significant sources of psychological distress. Moreover, we consider mindfulness intervention as a powerful strategy due to its feasibility because of its short duration and the short-term outcomes.

## Supplementary Information

**Additional file 1:** Cluster evaluation metrics.

**Additional file 2:** Sex distribution between clusters.

**Additional file 3:** Effect sizes between and within groups.

## Data Availability

The datasets used and analyzed during the current study are available from the corresponding author on reasonable request.

## Abbreviations

AC: Active Control Group
ANOVA: analysis of variance
Boot CI: bootstrapped 95% confidence interval
FFMQ: Five Facets of Mindfulness Questionnaire
MBI: Mindfulness-Based Intervention
MT: Mindfulness Training Group
NAS: Negative Affect Schedule
PANAS: Positive and Negative Affect Schedule
PAS: Positive Affect Schedule
PSS: perceived stress
SAI: State Anxiety Inventory
STAI: State and Trait Anxiety Inventory
SMS: State Mindfulness Scale
TAI: Trait Anxiety Inventory
