# Supplementary material for: Brief mindfulness-based training and mindfulness trait attenuate psychological stress in university students: a randomized controlled trial
Source: BMC Psychol. 2021 Feb 1;9:21. doi: 10.1186/s40359-021-00520-x (PMC7852130; doi:10.1186/s40359-021-00520-x)
Supplement: Supplementary file 1 — Additional file 1: Cluster evaluation metrics. [file 40359_2021_520_MOESM1_ESM.pdf]

Cluster evaluation metrics

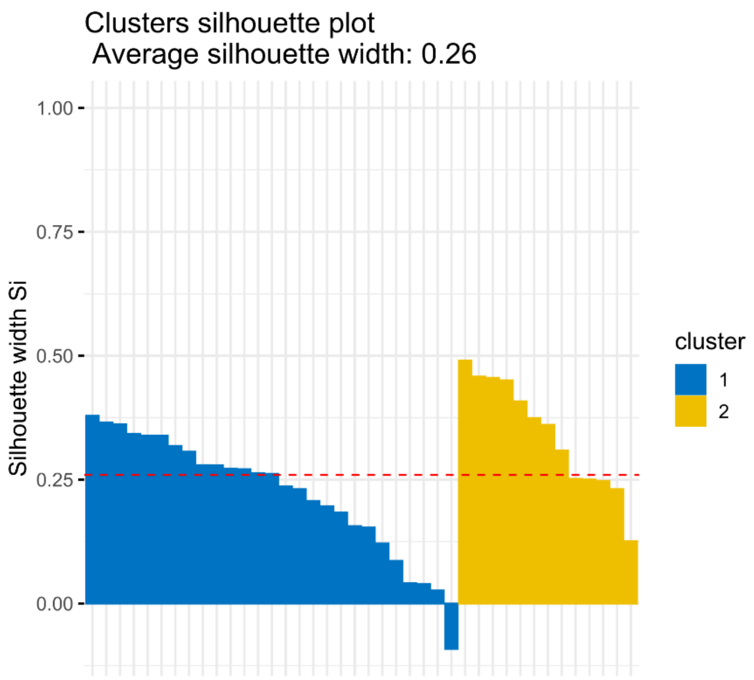

Fig. 1 Silhouette plot showing the individual silhouette width values ($s(i)$) and the only one negative value case in Cluster 1. Negative silhouette values may reflect outliers or individuals in the border of cluster. Average silhouette width is highlighted by the dashed red line. Details about $s(i)$ are showed below.

Table 1 Measures of cluster validation. Brief conceptualizations about them are given below.

Metric		Cluster 1	Cluster 2
Noise N	0		
Average between	20.33		
Average within	15.22		
WCSS		81.85	26.88
Silhouette width		.22	.34
Dunn	.12		
Entropy	.63		

- *Silhouette width*: defined as $s(i) = \frac{b_i - a_i}{\{(a_i),(b_i)\}}$, where *b* stands for distance between point *i* and other points in other clusters (clusters separation) and *a* stands for distance between point *i* and others in the same cluster (cluster cohesion), it estimates how well observations are clustered, where the closer to 1 the better clustered is the observation.

- *Noise N* = number of noise points in cluster.

- *Average between/within clusters*: average distance between and within cluster, where larger and smaller values indicate a well separated and cohesive cluster, respectively.

- *Within Cluster Sum of Squares (WCSS)*: the square sum of deviations from points to cluster center, it tells about cluster variability, where a small value is desirable; however, it is sensitive to number of points (larger samples have more variability).

- *Dunn*: measure of how compact and well separated the clusters are.

- *Entropy*: represents the amount of information, where the closer to 1 the better.
