# Supplementary material for: Brief mindfulness-based training and mindfulness trait attenuate psychological stress in university students: a randomized controlled trial
Source: BMC Psychol. 2021 Feb 1;9:21. doi: 10.1186/s40359-021-00520-x (PMC7852130; doi:10.1186/s40359-021-00520-x)
Supplement: Supplementary file 2 — Additional file 2: Sex distribution between clusters. [file 40359_2021_520_MOESM2_ESM.pdf]

Sex distribution between clusters

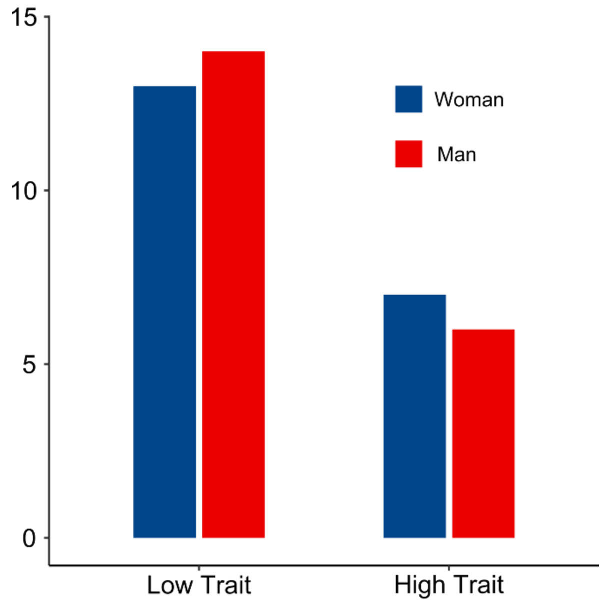

Distribution of sex (High Trait: 7 females, 6 males; Low Trait: 13 females, 14 males) showed to be similar into each cluster ($\chi^2 = 0$, p = 1).
