# Supplementary material for: Brief mindfulness-based training and mindfulness trait attenuate psychological stress in university students: a randomized controlled trial
Source: BMC Psychol. 2021 Feb 1;9:21. doi: 10.1186/s40359-021-00520-x (PMC7852130; doi:10.1186/s40359-021-00520-x)
Supplement: Supplementary file 3 — Additional file 3: Effect sizes between and within groups. [file 40359_2021_520_MOESM3_ESM.pdf]

Effect sizes between and within groups

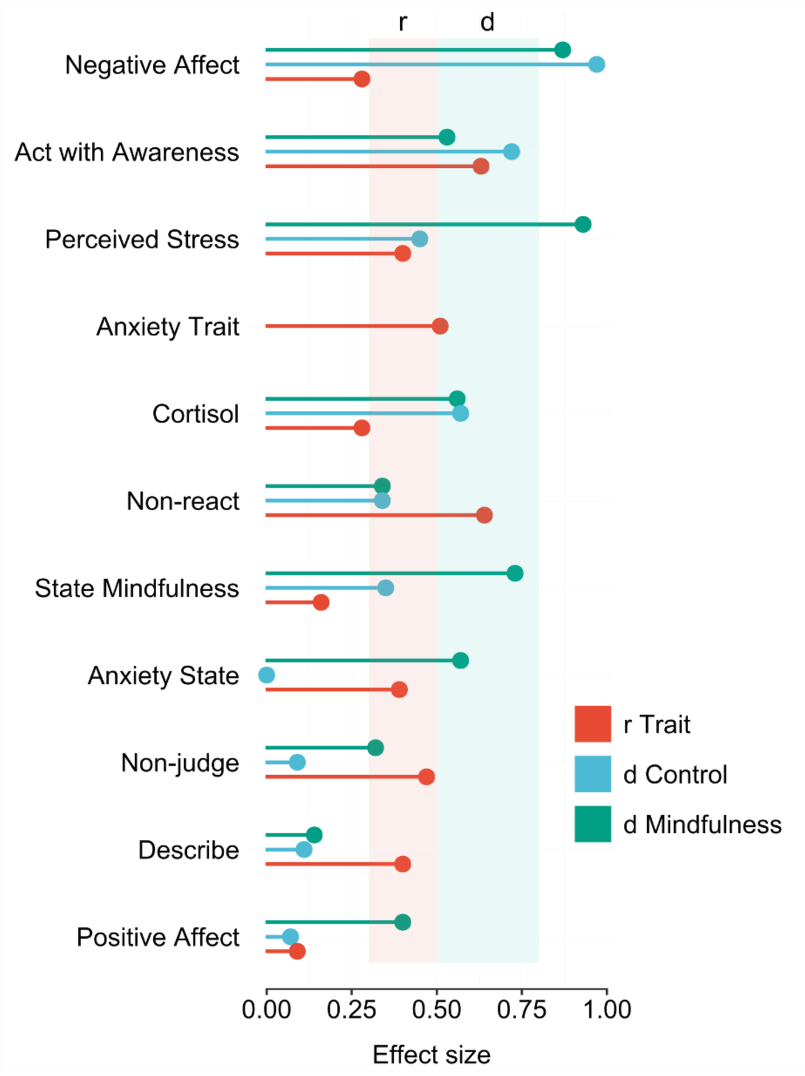

Summary of effect sizes for Wilcoxon sum rank test (*r*) between High and Low Mindfulness Trait (red) and for Mixed ANOVA (*d*) within Control (blue) and Mindfulness (green) groups. Shaded area in red denotes the region where *r* is considered as medium effect (upstream, small; downstream, high) while the region in green denotes medium *d* effect size (upstream, small; downstream, high).
